# Analysis of Discrimination Techniques for Low-Cost Narrow-Band Spectrofluorometers

**DOI:** 10.3390/s150100611

**Published:** 2014-12-30

**Authors:** Ismael F. Aymerich, Albert-Miquel Sánchez, Sergio Pérez, Jaume Piera

**Affiliations:** 1 Physical and Technological Oceanography Department, Institute of Marine Sciences (ICM-CSIC), Pg. Marítim de la Barceloneta, 37-49, Barcelona 08003, Spain; E-Mails: amsanchez@icm.csic.es (A.-M.S.); slezcano@gmail.com (S.P.); jpiera@icm.csic.es (J.P.); 2 AtlantTIC, University of Vigo (UVigo), Maxwell Street, Vigo 36310, Spain

**Keywords:** classification, denoising, fluorescence, low-cost sensors, normalization, signal processing, taxonomic discrimination, transformation

## Abstract

The need for covering large areas in oceanographic measurement campaigns and the general interest in reducing the observational costs open the necessity to develop new strategies towards this objective, fundamental to deal with current and future research projects. In this respect, the development of low-cost instruments becomes a key factor, but optimal signal-processing techniques must be used to balance their measurements with those obtained from accurate but expensive instruments. In this paper, a complete signal-processing chain to process the fluorescence spectra of marine organisms for taxonomic discrimination is proposed. It has been designed to deal with noisy, narrow-band and low-resolution data obtained from low-cost sensors or instruments and to optimize its computational cost, and it consists of four separated blocks that denoise, normalize, transform and classify the samples. For each block, several techniques are tested and compared to find the best combination that optimizes the classification of the samples. The signal processing has been focused on the Chlorophyll-a fluorescence peak, since it presents the highest emission levels and it can be measured with sensors presenting poor sensitivity and signal-to-noise ratios. The whole methodology has been successfully validated by means of the fluorescence spectra emitted by five different cultures.

## 1. Introduction

Chlorophyll (Chl) fluorescence techniques have been widely used to assess the taxonomic composition of microscopic photosynthetic organisms (phytoplankton) in order to avoid the time constraints imposed by the microscopic analysis of water samples [1]. The basis of fluorometric taxonomic discrimination relies in the specific features of the excitation and emission spectra of each phytoplankton taxonomic group [1,2], and multiple approaches have been used to determine such differences. For instance, the spectral deconvolution analysis, used in [3] to discriminate between two different organisms, or the self-organizing maps (SOM) technique, applied in [4] to classify seven strains from different taxonomic groups of phytoplankton, among others. Nevertheless, those techniques have mostly been tested with accurate and precise data obtained with expensive instruments. This involves an important limitation, since the observational costs spent in infrastructure and instruments in order to obtain high volumes of accurate data in shallow or open water is extremely high, and consumes most part of the money budget available in a research project. In this regard, the concept of “citizen science” has arisen as an effective methodology to mitigate the expenses while covering large areas with high temporal and spatial resolution measurements [5], but this concept only makes sense through the development of extreme low-cost sensors, as those presented in [6–12]. Reportedly, their accuracy (sensibility, resolution and signal-to-noise ratio (SNR)) is not comparable to the most precise (and consequently, expensive) alternatives, but they present a considerable potential if a correct pre-processing step is performed. Therefore, there is an increasing need for the development of signal-processing strategies able to suitably process the noisy and low-accurate data obtained from instruments based on low-cost sensors.

In this paper, the analysis of the discrimination skills of a potential low-cost hyperspectral fluorescence instrument presenting a lower performance in terms of sensibility, SNR and processing capabilities is presented. To this end, three different techniques based on pattern recognition are tested, evaluated and compared to find which one presents the optimal performance considering two main constraints. First, a successful taxonomic discrimination must be obtained even when using as primary information only the highest fluorescence emission levels (if the SNR of the sensor is extremely low, only those levels would be reliable), which correspond to the Chl fluorescence peak (around the 680 nm). This consideration differs from [3,4] where the whole optical spectra bandwidth is analyzed, and it is actually feasible assuming that the fluorescence signal in this wavelength range is not only due to the Chl-a emission peak, but also the Chl-b, -c and -d emission peaks along with additional complement pigments (such as the phycocyanin, whose fluorescence emission is located in the 630-to-660-nm band). Besides, this consideration relaxes the needed sensor's spectra bandwidth performance. Second, the computational cost needed to develop the algorithms must be optimally reduced in order to decrease the electronic hardware requirements needed to implement the instrument (which will directly influence on its economic cost). In order to deal with these two requirements and considering high levels of noise in the measurement samples, three signal-processing blocks previous to the classification one have been established, accounting for denoising, normalization and transformation of the measured data. The denoising block reduces the noise introduced by the sensor; the normalization block equals the emission contribution measured at different growth states, which improves the discrimination outcomes; and the transformation block transforms and reduces the data dimension, improving the computational-cost efficiency. Thereby, the most convenient technique in each of these three blocks, which, in combination with the best classification algorithm, provides an optimal taxonomic discrimination even when dealing with the two measurement constraints described above, is sought.

In order to test the performance of different algorithms in the presented signal-processing chain, the fluorescence spectra of five isolated cultures have been measured at different growth stages. Hyperspectral low-cost fluorescence instruments for *in-situ* or *in-vivo* measurements of phytoplankton responses have not been developed yet. Fluorescence sensors or instruments based on low-cost technology are presented in [6,10–12], but their measurements do not exhibit a hyperspectral performance. Therefore, measurements have been firstly obtained with an accurate fluorescence instrument and degraded afterwards in terms of resolution and SNR to emulate the potential low-cost sensor performance. Those measurements are then processed in each block, where well-known methods such as moving average, wavelet or principal components, are put into practice along with other algorithms developed in this study specifically designed for this work. This new approach, mainly based on a reliable signal-processing chain, considerably reduces the sensor's requirements (spectra bandwidth and computational cost) needed to perform a suitable classification. Besides, its conclusive results constitute an important stimulus to develop new and optimal low-cost fluorometers enhancing their discrimination capabilities and encouraging marine research groups to continue studying this field by considerably reducing the instrumentation costs.

This paper is structured as follows. A brief introduction to the algorithms used in this study is presented in Section 2. In Section 3, measurements from five phytoplankton cultures from different taxonomic groups are used to perform a comparison of the different algorithms. The results presented in this section were processed first with the original data, and later with a degraded version of the measurements in order to simulate the performance of a low-cost sensor. Section 4 outlines the conclusions derived from this work.

## 2. Processing Techniques

**Figure 1.**
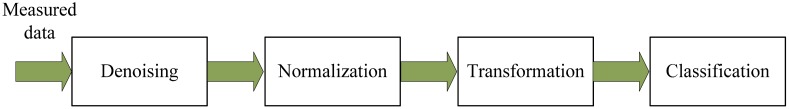
The four-step signal-processing chain.

Figure 1 shows the block diagram of the four-step signal-processing chain. Three steps before addressing a classification method, where the taxonomic discrimination is performed, are proposed in order to optimize the processing efficiency. Any electro-optical sensor is a noisy source mainly due to the shot and thermal noise, and this is emphasized in low-cost sensors, which usually present a lower performance. Denoising techniques are firstly applied to mitigate the noise effect, considering that a careful attention must be paid in order to avoid the loss of information due to an excessive smoothing. The fluorescence intensity depends upon the cell concentration, the biological growth state, the temperature conditions and the incident light, among other factors, and measurements of the same culture may present significant range variations. Since the classification techniques are usually based on the Euclidean distance between the sample under test and a reference, their objective functions will not appropriately discriminate the samples if such variations are presented within the same culture. Therefore, all measurements must be normalized in a second step in order to make the contribution of their particular features equivalent. Finally, the transformation techniques that adapt the data to increase the discrimination capacity of the classification algorithms, and the reduction of dimension methods that increase the efficiency of the learning algorithms, are included in the third step. In the latter, if the classification techniques have to deal only with those wavelengths that are more representative of the features that characterize the culture (obviating redundant information), the computational cost is considerably reduced.

The whole set of techniques used in each step are presented in Table 1, and described in the following subsections. Widely known methods such as moving average, principal components or *k*-neighbors are used along with other techniques developed and adapted to improve the taxonomic analysis proposed in this paper. Moreover, the complete signal-processing chain has been centered in the Chl-a fluorescence peak (around 680 nm), which largely simplifies the computational cost that the analysis of the whole hyperspectral data would need.

### 2.1. Denoising

Optical detectors are subjected to several influences such as optical shot noise (which follows a Poisson distribution), thermal noise (Poisson distribution), read noise (approximately Gaussian), background light from blackbody radiation (Plank distribution), flicker noise (pink power distribution) and technical noise due to various imperfections (which do not follow a specific distribution). The noise-floor level in a measurement is determined by the thermal and the read noises, while the shot noise dominates at high signal values. In a low-performance sensor, it is expected to have significant levels of noise and, in consequence, a poor SNR. Therefore, a denoising block is needed as a first step for the proposed processing chain. Three different techniques have been considered to smooth the measurements acquired for this study (see the first column of Table 1). These techniques are briefly described below.

#### 2.1.1. The Weighted Moving Average Method

The weighted moving average (WMA) [13] is the most widely used technique for denoising. In it, the output averaged data vector (*y*) can be computed as the weighted mean of the nearest 2 · *P* wavelengths (*P* wavelengths for each side) for each value of the noisy raw data (*x*), and can be expressed as:

(1) $$y\left(\lambda \right)=\frac{1}{\sum_{\rho =-P}^{P}\omega \left(\rho \right)}\sum_{\rho =-P}^{P}\omega \left(\rho \right)x\left(\lambda -\rho \right)$$

being *ω* the weighting factor vector and *λ* the wavelength. The particular case where all weighting factors are equal to one is usually known as the standard moving average.

#### 2.1.2. The Savitzky-Golay Method

The Savitzky-Golay technique [13] computes a local polynomial regression to approximate the nearest noisy samples using the least squares method, as:

(2) $$y\left(\lambda \right)=\sum_{\rho =-P}^{P}b_{0}\left(\rho \right)x\left(\lambda -\rho \right)$$

being *b*_0_ the steady-state Savitzky-Golay filter which coefficients are determined using the least-squares fit. The main advantage of this approach is that it tends to preserve distribution features such as relative maxima, minima and width, usually flattened with the WMA technique at the expense of not removing as much noise as the WMA.

#### 2.1.3. The Wavelet Method

The wavelet denoising [14] is a more refined method that separates the frequency content of the original signals into different data structures. The low-frequency components (approximation coefficients) keep the global features of the signal, while the high-frequency components (detail coefficients) retain the local features. For discrete data, it can be computed as:

(3) $$\tilde{x}\left(\lambda ,\kappa \right)=\sum_{\rho =-\infty }^{\infty }x\left(\rho \right)\frac{1}{\sqrt{2^{\lambda }}}\Psi \left(\frac{\rho -\kappa 2^{\lambda }}{2^{\lambda }}\right)$$

being *ψ* the mother wavelet function, *x̃* the discrete wavelet transform (DFT), and *κ* a location parameter. A fast algorithm to compute the discrete wavelet transform is presented in [15]. Soft and hard threshold techniques [16,17] can be used to reduce the noise, and the threshold level is selected as [16]:

(4) $$\text{thr}=\xi \sqrt{2\log\left(n\right)}$$

where *n* is the number of samples and *ξ* is a rescaling factor estimated from the noise level present in the signal. The estimation of the noise level can be based on the first level of the detail coefficients (*D*_1_) as [14]:

(5) $$\xi =\frac{\text{median}\left(\left|D_{1}\right|\right)}{0.6745}$$

Finally, by applying the inverse wavelet transform, a smoothed version of the original signal is recovered. The advantage of this method relies on a denoising procedure that does not affect the sharp structures of the original data.

**Figure 2.**
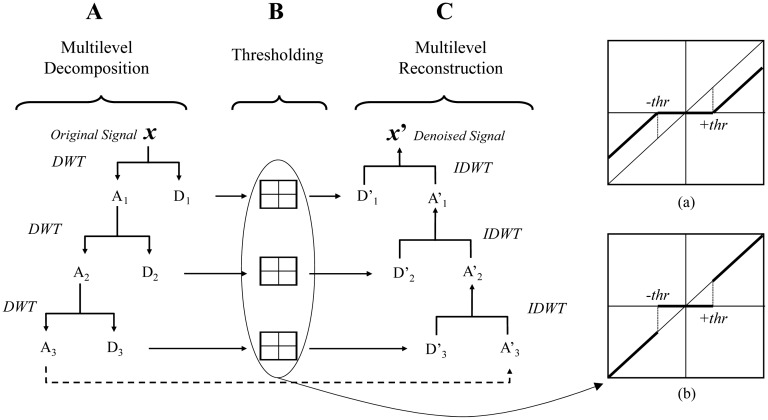
Schematic diagram of the three steps of the wavelet method: multilevel decomposition, thresholding, and multilevel reconstruction. Thresholding is obtained *via* (**a**) soft threshold techniques or (**b**) hard threshold techniques.

**Figure 3.**
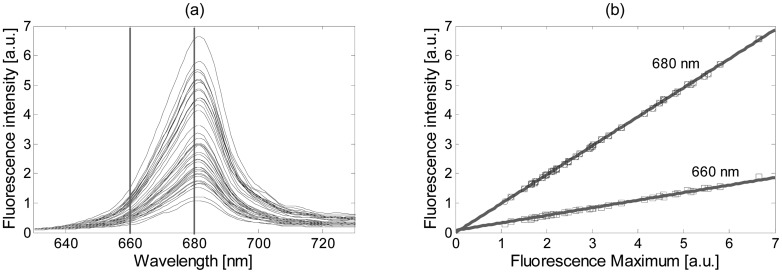
(**a**) Fluorescence measurements of a particular culture at different growth states; (**b**) Fluorescence at 660 nm and 680 nm related to each fluorescence maximum, and linear regression for the two cases.

Figure 2 shows an example of a three-level wavelet decomposition. First, the original signal *x* yields one series of approximation coefficients *A*_3_ and a set of three distinct detail coefficient signals *D*_1,2,3_. Then, either a soft or a hard threshold methodology is applied on the detail coefficients. In a soft threshold (Figure 3a), coefficients smaller than the threshold ‘*thr*’ are suppressed while the rest of the coefficients are shrunk an equivalent of the threshold value [15]. In a hard threshold (Figure 3b), coefficients smaller than the threshold ‘*thr*’ are set to 0 while the rest of the coefficients remain intact. The denoised profile *x′* is finally recovered from the transformed coefficients by applying the inverse discrete wavelet transform (IDWT).

### 2.2. Normalization

Once the measurements have been denoised, the next step is the normalization. The second column of Table 1 shows the normalization methods considered in this paper and described below.

#### 2.2.1. The Min-Max Method

The Min-Max is a simple method of fitting the fluorescence curve into a fixed range. Minimum and maximum values (*a* and *b*, respectively) become equal for all the samples, and the normalized curve is obtained with:

(6) $$y\left(\lambda \right)=a+\frac{\left(x\left(\lambda \right)-\min\left(x\right)\right)\left(b-a\right)}{\max\left(x\right)-\min\left(x\right)}$$

#### 2.2.2. The Growing Spectra Modeling Method

The growing spectra modeling (GSM) is a new method that exploits the simplicity of the Min-Max normalization but uses the information of all the values at each wavelength simultaneously in order to increase its robustness. In it, each wavelength fluorescence value of a particular culture and at a specific growth state is compared with its fluorescence maximum. Measurements on different cultures have shown that this relationship is linear at all wavelengths, which allows obtaining an accurate approximation using their linear regression coefficients, as:

(7) $$Fl_{\lambda }\left(m\right)=a_{\lambda }m+b_{\lambda }$$

being *a_λ_* and *b_λ_* the linear regression coefficients of the wavelength *λ*, and *Fl_λ_* its fluorescence evaluated at *m*. Figure 3a shows an example of the smoothed fluorescence measurements of a particular culture at different growth states. As can be observed, the fluorescence maximum presents an important level variability according to different growth states. When all the measurements on a single wavelength are plotted against its fluorescence maximum, a linear relationship, as shown in Figure 3b for two particular wavelengths (660 nm and 680 nm), is obtained. The linear regression has also been plotted for these two cases, showing a decrement on the slope when moving away from the maximum. In general, a unitary slope is obtained around 680 nm, and a close-to-zero slope around the fluorescence minimum. The model finally uses the linear regression coefficients to compute the normalization factor for each measurement and wavelength. This is done by evaluating Equation (7) at two point values, the maximum fluorescence in that measurement (obtaining *Fl_λ_*_1_) and the desired (or normalized) maximum fluorescence (obtaining *Fl_λ_*_2_). The coefficient obtained from the relationship *Fl_λ_*_2_/*Fl_λ_*_1_ is the normalization factor used to normalize the initial data value.

#### 2.2.3. The Standard Normal Variate Method

The standard normal variate (SNV) [18,19] is a robust method against noisy data. It is based on the mean and variance (*μ* and *σ*^2^, respectively) matching of all the measured samples, as:

(8) $$y\left(\lambda \right)=\frac{\left(x\left(\lambda \right)-\mu +\mu_{\text{tot}}\right)\sqrt{\sigma_{\text{tot}}^{2}}}{\sqrt{\sigma^{2}}}$$

being *μ_tot_* and $\sigma_{\text{tot}}^{2}$ the averaged mean and variance of the whole set of samples. A typical approximation is done considering *μ_tot_* =0 and $\sigma_{\text{tot}}^{2}=1$.

#### 2.2.4. The Modified Scale-Based Normalization Method

The three previous methods significantly distort those signals that are more different from the general pattern, leading, in some cases, to a significant deformation of the small details that characterize the nature of the sample. A more general and flexible version of the SNV method is the scale-based normalization (SBN) introduced in [18]. When applying the wavelet decomposition to a signal, its variance is also faithfully decomposed, allowing a more precise scaling. Variance normalization to 1 ( $\sigma_{\text{tot}}^{2}=1$) and mean to 0 (*μ_tot_* = 0) is performed using only those wavelets that do not contain the high-frequency noise, as:

(9) $$y\left(\lambda \right)=\frac{D_{i}\left(\lambda \right)+\cdots +D_{j}\left(\lambda \right)}{\sqrt{\sigma_{i}^{2}+\cdots +\sigma_{j}^{2}}}$$

being *D_j_*, …, *D_j_*(*λ*) the *j* − *i* noise-free detail functions of the wavelet decomposition (obtained with Equation (3)), and $\sigma_{i}^{2},\cdots ,\sigma_{j}^{2}$ their variances. The SNV method constitutes a special case of Equation (9) by using *i* = 1 and *j* = *log*_2_*n* (being *n* the number of discrete points of the original signal).

**Figure 4.**
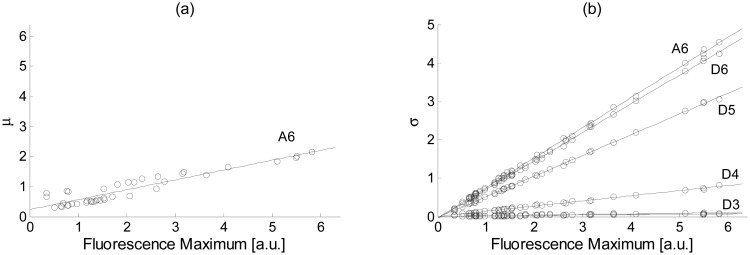
(**a**) Mean (*μ*) plotted against fluorescence maximum for the approximation coefficients at level 6, and (**b**) standard deviation (*σ*) plotted against fluorescence maximum for the approximation coefficients at level 6 and detail coefficients at levels 3–6, all obtained from fluorescence measurements on an actual phytoplankton culture. The linear regression is also shown for all cases.

In this paper, this method has been extended and the normalization of the mean and variance in the approximation coefficients case, and only the variance in the detail coefficients case (since their mean is zero), is done using normalization coefficients different from 0 and 1, respectively. Thereby, the information useful for further classification contained in the relationship between the different levels is conserved. In order to select suitable normalization coefficients, the relationship between the mean and the standard deviation of the approximation and detail coefficients and their fluorescence maximum is firstly obtained. Several measurements on different cultures have shown that this relationship is linear, as seen in the example of Figure 4. In the standard deviation case (Figure 4b), the relationship is extremely linear with a decreasing slope as the detail level decreases. A higher dispersion is obtained in the mean case (Figure 4a), even though a linear relationship can still be considered. Then, the linear regressions for all plots are obtained as it was done with Equation (7) (also shown in Figure 4), and they are evaluated at the desired (or normalized) maximum fluorescence (*μ_D_* and *σ_D_*). Finally, each wavelet level is independently normalized using (8) with $\sigma_{\text{tot}}^{2}=1$ and *μ_tot_* = 0, and adjusted as:

(10) $$A/D_{\text{iSMB}}\left(\lambda \right)=A/D_{\text{iSNV}}\left(\lambda \right)\cdot \sigma_{Di}+\mu_{Di}$$

being *A/D_i_* the *ith* approximation/detail coefficient.

### 2.3. Transformation and Reduction of Dimension

Before addressing the classification problem, the transformation and dimensionality reduction step is presented. The third column of the Table 1 shows the different approaches proposed in this paper. A brief description of these techniques is presented below.

#### 2.3.1. The Derivative Method

The derivative method [20] is a transformation method that computes the derivative of the signal for discrimination purposes. A suitable analysis of this derivative is able to highlight subtle features from the original spectra.

#### 2.3.2. The Genetic Algorithm Method

The genetic algorithm [21] is a heuristic-search method used to estimate those wavelengths that are more representative of the significant features that characterize a culture in order to reduce the data dimension. It is based on the process of natural selection and exploits the principles of evolution to find the optimal results. Its performance can be summarized as follows. First, a vector of solutions (each solution contains a reduced number of wavelengths) is randomly generated. Then, the complete vector is evaluated by the fitness function. This is done by using the fluorescence information contained only in those wavelengths given by each solution in a classification technique and verifying if a suitable discrimination is obtained. Better results give a better score to that solution.

**Figure 5.**
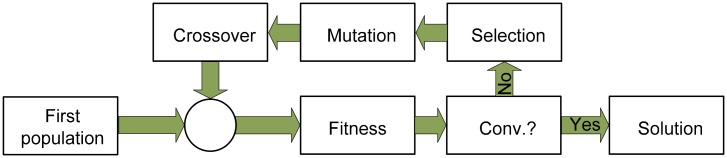
Block diagram of the genetic algorithm performance.

After the evaluation, the algorithm may stop if either a maximum number of generations (each generation is a new vector of solutions) or a satisfactory fitness level has been reached. If the convergence condition is not fulfilled, the best solutions are selected and separated. Part of these elite is then recombined (crossover) and randomly mutated to provide genetic diversity and broaden the search space. The new set of solutions is reevaluated and inserted again into the solutions' vector, which completes the cycle. After convergence is achieved, the algorithm presents the best solution it has been able to find. Figure 5 summarizes the performance of the genetic algorithm.

#### 2.3.3. The Principal Component Analysis Method

The principal component analysis (PCA) method [22] is an unsupervised method that uses an orthogonal transformation to convert the covariance matrix of the measured data into a set of linearly uncorrelated variables called principal components. This method highlights the similarities and differences between measurements and allows to clearly discriminating the least significant components, which can be discarded reducing thus the number of dimensions without much loss of information.

### 2.4. Classification

Classification algorithms can be grouped into parametric and non-parametric techniques. For parametric classifiers the data are assumed to follow a statistical distribution, which may be a major drawback if the data do not meet this condition. Furthermore, these algorithms are more likely to suffer from the problem of the curse of dimensionality or Hughes phenomenon [23] in hyperspectral classification. Therefore, only non-parametric methods for taxonomic discrimination are shown in the fourth column of Table 2. They are described below.

#### 2.4.1. The *k*-Neighbors Method

The *k*-neighbors method [24] is a nonparametric technique that computes the distance between the sample that needs to be classified and a known set of *k* samples. The sample is simply assigned to the class of the nearest neighbors.

#### 2.4.2. The SOM Method

The SOM method [4] consists of an artificial neural network based on unsupervised learning, *i.e.*, the network learns only based on the training data. Each neuron has a weighting vector with the same dimension as the input data. The SOM projects the high-dimensional input samples onto a two-dimensional map, a feature useful for their visualization and classification, and modifies the weighting vector of those neurons closer to the sample. This is done as:

(11) $$W_{ij}\left(\lambda +1\right)=W_{ij}\left(\lambda \right)+\alpha \left(\lambda \right)\times h_{c}\left(\lambda \right)\times \left(x\left(\lambda \right)-W_{ij}\left(\lambda \right)\right)$$

where *x*(*λ*) is the input data vector, *h_c_*(*λ*) is the learning neighborhood function (typically a Gaussian bell-shaped function), and *α*(*λ*) is the learning rate. At the end of the training phase the map is organized such that neighboring neurons in the grid have similar weighting vectors.

#### 2.4.3. The Growing Cell Structure Method

The growing cell structure (GCS) method [25] is a self-organizing network which important feature is its ability to automatically find an optimal network structure and size suitable to deal with a specific problem. The algorithm starts with a very simple network and inserts new neurons near those positions that match better with the input data. This controlled growing process is an important advantage over other static neural networks such as SOM, which initiates the training process using a regular network with a fixed number of neurons, and may not be able to suitably adapt their initial structure to the problem under analysis.

## 3. Results and Discussion

In order to test the four-step signal-processing chain proposed in Section 2, the fluorescence emissions of five cultures belonging to different taxonomic groups were measured using an Aminco-Bowman Series 2 luminescence spectrometer (configured with a 4-nm slit width and a scan speed of 20 nm/s) (see Table 2). In all cases, the excitation wavelength was centered at 470 nm (since this excitation wavelength allows an optimal classification, as shown in [4]), and an emission bandwidth between 200 nm and 800 nm in steps of 1 nm was obtained. Successive daily measurements were acquired while the cultures kept alive. Only some initial measurement samples were discarded while the concentration of phytoplankton was too diluted to obtain a meaningful signal with the spectrofluorometer (the first useful measurement is different for each culture due to a different growth speed among them). The total number of measurements is shown in Table 2. This experiment was done twice (first and second sampling) to increase the number of measurements and obtain a dataset useful for a suitable classification.

In this section, each algorithm described above is analyzed and compared with the others in the same chain step in order to determine its effectiveness for a suitable classification. To this end, the signal processing has been applied only on the 630-to-730 nm band, avoiding the thermal emission (beyond the 730 nm) and the Rayleigh and Raman scatterings (below the 630 nm), with the Chl-a fluorescence peak at the band center. Thus, by avoiding the full measured spectra, the computational cost needed to suitably process the signal is largely simplified. At the end of this section, the algorithms are tested again with a degraded version of the measurements shown in Table 2 (by reducing the spectra resolution and adding Gaussian noise), emulating the performance of worse sensors. Both methods (reduction of the emission band and measurement degradation) have been applied to determine if the algorithms presented in this paper are suitable for taxonomic classification when measuring with low-quality sensors and instruments.

### 3.1. Denoising

Smoothing methods cause changes to the original spectral data that may lead to inaccurate results in subsequent methods if relevant signal particularities are eliminated along with noise. In order to objectively examine the statistical properties of the measured data processed with the three denoising methods proposed in this paper, the covariance matrices of the original and smoothed data, which show the variance relationship between different wavelength distributions, are compared. The more similar the matrices are the least distortion is being introduced by the denoising method.

**Figure 6.**
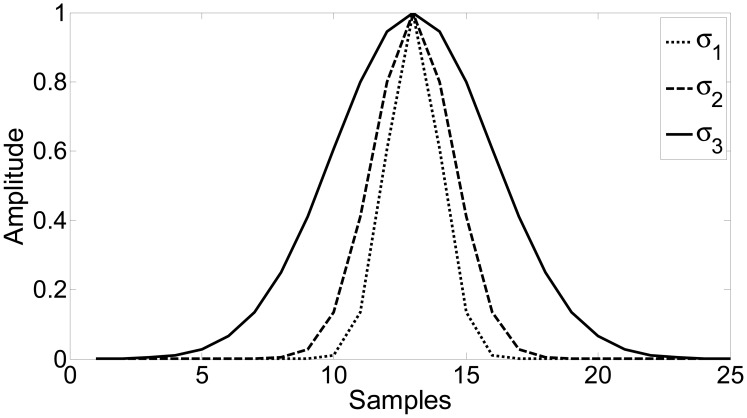
Gaussian windows used with the WMA method.

Table 3 resumes the results obtained from fluorescence measurements in the Duna culture case, using different conditions for each denoising method. The similarity between the covariance matrices of the original and smoothed data is evaluated using the root mean square error (RMSE). As can be seen, the WMA method has been applied along with square windows of 3, 7 and 11 samples and with three Gaussian windows, each one with a different standard deviation (*σ*_1_, *σ*_2_ and *σ*_3_, respectively), as shown in Table 3 and Figure 6; the Savitzky-Golay method has been applied along with windows of 13, 17 and 23 samples; and a 6th-level wavelet method (using the Daubechies wavelet family with nine vanishing moments as in [14] and suitable for signals with zero-mean Gaussian white noise) has been applied considering two different filtering thresholds. While the first method (*thr*_1_) uses a soft threshold on the whole detail level set, the second method (*thr*_2_) applies a hard threshold on the same set (Figure 3a and 3b, respectively). In both cases, the adaptive threshold selection technique described in Section 2.1.3 is used to estimate the suitable threshold level. It must be noted that the optimal mother wavelet mainly depends on the noise properties and the signal characteristics, and therefore, once the low-cost sensor is implemented, an analysis of its characteristics should be performed for a suitable selection.

In general, for small or intermediate values of the smoothing parameters, the covariance curves present a high overlapping. As the smoothing factor increases, both curves diverge, increasing the RMSE between them. As observed, the covariance matrix comparison shows that the statistical properties in the WMA case diverges for square or Gaussian windows wider than a few samples, while the Savitzky-Golay method keeps a small distance length even for windows up to 17 samples. Finally, the wavelet method shows a smaller distance in the hard threshold case than in the soft one. Similar results have also been obtained using different cultures. Table 3 gives an idea about the smoothing rate introduced by each algorithm, but it is not decisive when selecting the most suitable one. Further results are shown in Section 3.4 when using them to classify the samples.

**Figure 7.**
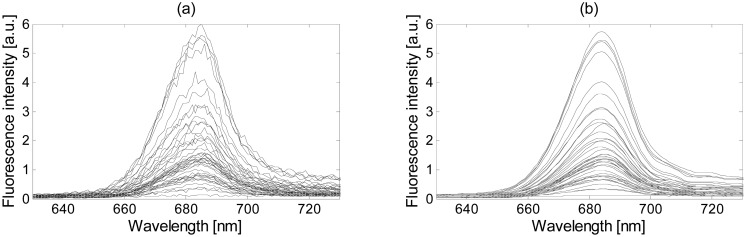
(**a**) Original and (**b**) smoothed spectra of all the Pl measurements obtained with the Savitzky-Golay method and *n* = 17.

Figure 7 shows the original and smoothed spectra for the Pl measurements using the Savitzky-Golay method and *n* = 17 as an example.

### 3.2. Normalization

**Figure 8.**
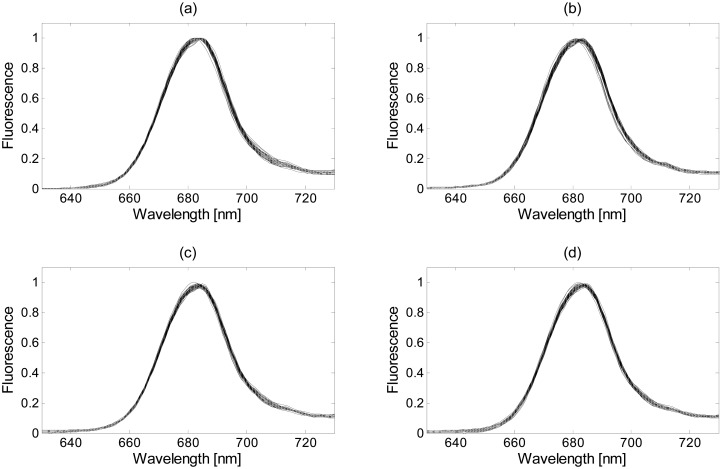
Normalizations applied on the denoised Thwi measurements (with wavelet method and soft threshold) using: (**a**) the min-max method; (**b**) the growing spectra modeling (GSM) method; (**c**) the standard normal variate (SNV) method; (**d**) the modified scale-based normalization (SBN) method.

Figure 8 shows the four normalization methods proposed in this paper applied on the Thwi measurements after denoising using the wavelet method and soft threshold.

As can be observed, the four spectra plots are quite similar, but slight differences can be appreciated. The spectra obtained with the min-max method, Figure 8a, presents flat shapes around the 640 nm and 680 nm with minimum and maximum values, respectively, not seen in any other plot, due to the scaling method. Such distortion may affect the statistical properties present at those wavelengths. As expected, the GSM method improves the spectral shape, as shown in Figure 8b, and presents the best normalization below 660 nm. However, all the curves tend to concentrate around a single point in its maximum since it is taken as the reference and it can affect the classification step. The SVN and the modified SBN methods, Figure 8c and d, respectively, present similar curves and do not suffer from any distortion on their fluorescence values. The four algorithms are objectively compared in Subsection 3.4 against the denoising and classification methods to determine the most suitable one.

### 3.3. Transformation and Reduction of Dimension

#### 3.3.1. The Derivative Method

**Figure 9.**
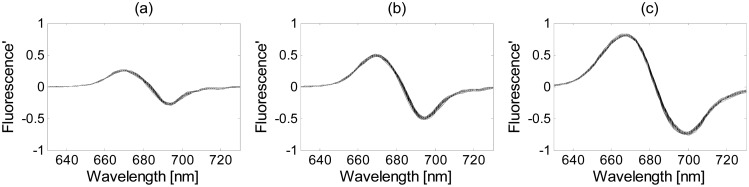
Derivative of the Thwi samples for different band separation: (**a**) 5 sampling intervals; (**b**) 10 sampling intervals; (**c**) 20 sampling intervals.

The derivative of the denoised and normalized samples (using the wavelet and SBN methods, respectively) of the Thwi culture was obtained using different band separations, as shown in Figure 9. As the band separation increases, a smoother curve is obtained. In order to know if the derivatives of the original fluorescence signals contain hidden properties that may facilitate the discrimination process, they are used in Subsection 3.4, along with the three classification methods, to compare its taxonomic discrimination results with the ones obtained using the original measurements.

#### 3.3.2. The genetic algorithm method

The first step before using the genetic algorithm is to find the minimum data dimension that keeps a suitable classification efficiency. The maximum likelihood estimator (MLE) technique [26], which uses the principle of maximum likelihood on the distances between close neighbors to group them, was applied on the fluorescence data of the five cultures denoised and normalized with the wavelet (hard threshold) and SBN methods, respectively, obtaining a minimum dimension of 5 bands. Then, the genetic algorithm was used in combination with the *k*-neighbors classification method (with *k* =1) to find the value of these five wavelengths. Among the classification methods, the *k*-neighbors was selected since it does not need a training and thereby it is the fastest one. The results obtained after 20 generations over an initial vector of 100 solutions are 637 nm, 677 nm, 694 nm, 710 nm and 720 nm. Since these results are spaced along the whole bandwidth, it can be concluded that the particular features of each culture are not concentrated in a narrow band but widely distributed.

#### 3.3.3. The PCA method

**Figure 10.**
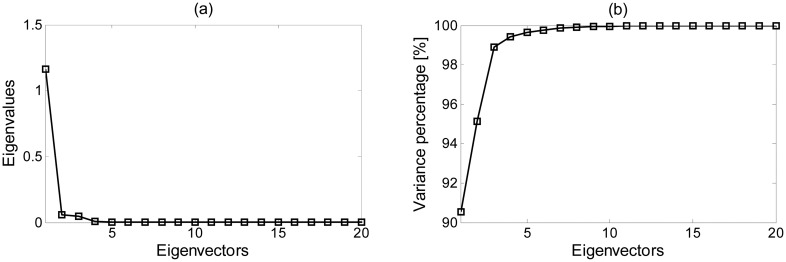
Representation of (**a**) the first 20 eigenvectors, and (**b**) their percentage of variance.

Figure 10 shows the results obtained with the PCA method applied on all the samples. As can be seen, a significant reduction of the data dimension can be applied since the first three components concentrate the 99% of the data variability. The other ones will not significantly contribute to obtain a better classification of the culture. In the next subsection, the first three components obtained with this algorithm are used in combination with the three classification methods to compare its results with previous methods.

### 3.4. Classification

The comparison between the classification methods (and the different denoising and normalization techniques) has been performed using the confusion matrix and the Kappa index (*K*) [4]. The confusion matrix displays both the number of samples that were correctly and incorrectly classified, and, in the latter case, provides insight into which was the wrong chosen culture. The Kappa index is a measure of the global classification error whose calculation is made from elements of the confusion matrix.

In order to maximize the performance of *k*-neighbors, a previous optimization of the variable *k* was done finding the value that presents the best classification results, obtaining *k* = 1. The training for the SOM and GCS classification algorithms, with 32 nodes each, was done using both sampling columns of Table 2 in a 5-fold cross-validation technique.

Tables 4, 5 and 6 resume the classification performance of the three classification methods, in combination with the three denoising methods and the four normalization techniques, through the Kappa index. As can be observed, all combinations present accurate classifications achieving in some cases a perfect result. In general, the net growing concept seems to present a better performance than the static net of SOM. However, the three tables coincide in pointing out the *k*-neighbors as the best classification method. Besides, among the denoising techniques, the WMA denoising algorithm (Table 4) gives the higher Kappa indices, as the SNV does among the normalization ones. Therefore, an optimal solution for the signal-processing chain is obtained when using these two algorithms (WMA and SNV) in combination with the *k*-neighbors method.

Table 7 shows the Kappa index obtained with the three classification methods and the three transformation methods when considering the WMA (Gaussian window with *σ*_2_) as a denoising method and the modified SBN to normalize. The optimal performance is obtained again with the *k*-neighbors method using only the five bands given by the genetic algorithm (as expected since the genetic algorithm uses the *k*-neighbors method to find the suitable wavelengths) or in combination with the first three components given by the PCA method. SOM gives its best result using the five bands of the genetic algorithm and, in contrast, GCS gives its one in combination with the PCA.

All these results reinforce the idea that an important reduction of the data dimension can be applied without much loss of performance, which involves a reduction of the needed computational cost. In order to evaluate the degree of optimization, Table 8 shows the time employed on the execution of the previous example with and without a reduction of the data dimension using an Intel Pentium 4 at 3 GHz, with a 1 GB RAM and running a Windows 7. As can be seen, the time needed to complete the signal processing is reduced between 30%–33% in the SOM case, 8%–9% in the GCS case, and 20%–24% in the *k*-neighbors case. Even without the further computational-cost reduction, the *k*-neighbors algorithm is already much optimal than SOM and GCS algorithms, since training is not necessary, and therefore preferred from this point of view. On the other hand, the derivative of the original spectra does not seem to improve in a significant way the classification techniques, and its performance worsens proportionally to an increasing derivative order.

Tables 9 and 10 show the averaged confusion matrices (due to the five-fold cross-validation) for the worst solutions obtained in Tables 4, 5, 6 and 7, that is, the one obtained with the SOM classification method when using the Savitzky-Golay and the Min-Max methods to denoise and normalize (Table 9), and the one obtained with the SOM algorithm when using the wavelet method with a soft threshold and the Min-Max method to denoise and normalize (Table 10). Thus, those samples that are more difficult to be suitable classified can be identified. In the Savitzky-Golay case (Table 9), some Pl samples are classified as Duna, but the major error is produced with the Iso samples classified as Amin. In the wavelet case (Table 10), some Duna samples are classified as Pl, but, again, a considerable error is produced with the Iso samples classified as Amin. Both results show that small similarities exist between Duna and Pl samples and an important likeness between Iso and Amin.

### 3.5. Classification Using Degraded Samplings

**Figure 11.**
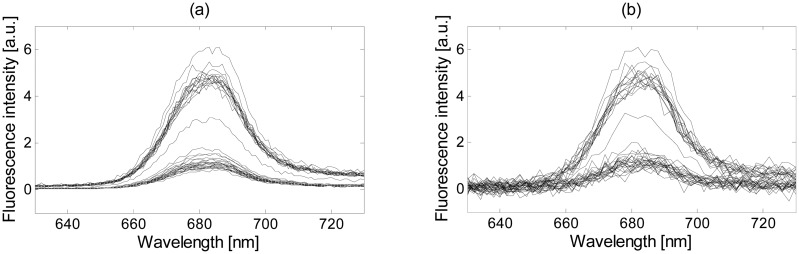
(**a**) Measured Thwi fluorescence; and (**b**) Thwi fluorescence after reducing the spectral resolution and adding noise, which follows a white Gaussian distribution with zero mean and a variance of 0.03, to emulate the measurements obtained with low-cost sensors.

The fluorescence measurements used in the previous subsections were obtained with an accurate spectral resolution of 1 nm and using a slit width of 4 nm. In order to simulate the performance of a low-cost fluorometer, the signal quality was degraded by reducing its spectral resolution by a factor of 2 (since the monochromatic filter bandwidth was of 4 nm, no loss of information is produced) and its SNR by adding noise. Since the measurement's noise-floor level is mainly described through a white Gaussian distribution, as stated in Subsection 2.1, the added noise followed a white Gaussian distribution with zero mean and a variance of 0.03. Figure 11 shows an example before and after this signal degradation.

The algorithms of the signal-processing chain described in this paper were applied on the degraded samplings to determine if a suitable classification performance could be obtained under such constraints. The WMA denoising method with a Gaussian window (and using *σ*_2_) showed to be the most successful one, as seen in Tables 4, 5 and 6. Therefore, this method was selected to compare the three classification methods against the four normalization techniques, as seen in Table 11. Again, the best result is obtained when using the SNV normalization method and the *k*-neighbors classification one, achieving almost a perfect classification.

In order to determine if this new set of samples can be dimensionally reduced to decrease the computational cost but still obtaining accurate results, the genetic algorithm was applied again to obtain the five frequencies that mostly characterize them. By firstly using only these five frequencies along with the *k*-neighbors classification method, and secondly the PCA along with the *k*-neighbors classification method, the Kappa index obtained were of 0.890 and 0.981, respectively. This classification result shows that the best combination of algorithms, in the *k*-neighbors method case, include the PCA method as the optimal one to reduce the computational cost (the successful classification percentage only drops a 2% and thus an accurate classification result is still obtained, whereas the execution time is reduced by a 28%). Additionally, in the PCA case, a study of the classification performance for different levels of noise was performed by modifying the variance of the Gaussian distribution, obtaining the results shown in Table 12. As expected, the Kappa index decreases as the variance increases. At a variance beyond 0.2 the classification cannot be considered successful (Kappa index drops far below 0.8).

**Figure 12.**
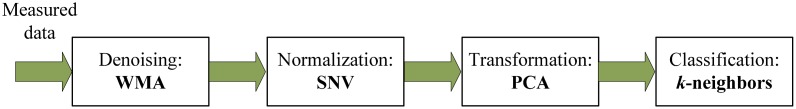
Optimal algorithms for the four-step signal-processing chain designed to deal with low-accurate narrowband fluorescence measurements.

The results presented above show that the best combination of algorithms in the signal-processing chain that showed an optimal classification with the accurate fluorescence measurements, is also the best combination when handling with low-accurate data (degraded in terms of resolution and SNR). As stated in Table 11, this combination includes the WMA method to denoise, the SNV method to normalize, the PCA method for a dimensional reduction and the *k*-neighbors to classify the measurements (see Figure 12), reaching a discrimination performance with a Kappa index of 0.981 (when *σ* = 0.03). This is also the best solution in terms of computational cost, since the *k*-neighbors algorithm presents the lowest execution time (5% of the total SOM execution time and 1% of the whole GCS execution time), and, after adding the dimensional reduction block, the execution time is further reduced by a 24% (as seen in Table 8).

Taking into account the results presented above, it has been proven the initial hypothesis of a feasible taxonomic classification using only the narrow 630-to-730 nm band of fluorescence emissions, which corresponds to the Chl-a peak, since other cellular contents (which differs depending on the strain) also modify its spectral shape. With this, the two constraints given by the measurements obtained with low-performance sensors, *i.e.*, high noise levels in a narrow bandwidth and an optimal computational cost, have been dealt. To conclude, despite the fact that a limited number of samples obtained from only five different cultures constituted the whole dataset, the results presented in this section are significantly accurate, and constitute an optimistic beginning to continue working in this direction, either designing more accurate algorithms or improving the current ones. Besides, they stimulate the investment in the development of a new hyperspectral low-cost sensor with discrimination capabilities centred in the Chl-a peak spectral range. It should be noted that, if a new low-cost sensor was actually developed, its measurement uncertainty (regarding to spectrometric and radiometric errors) should be perfectly characterized and evaluated to see if the performance of the signal processing presented in this paper is severely degraded.

## 4. Conclusions

Current constraints in money budget for research projects have motivated the development of new low-cost technologies. That, in consequence, requires an effort to extract as much information as possible from instruments exhibiting a low performance. In this paper, an optimal-computational-cost signal-processing chain designed to deal with fluorescence measurements featuring poor signal-to-noise ratios, low resolution, narrow bandwidth and, therefore, suitable for low-cost sensors and instruments, has been presented. The main objective of this research was focused in finding that combination of algorithms that optimizes the instrument performance when discriminating between different taxonomic cultures of phytoplankton species present in marine environments considering two constraints. The first one was given by the potential low-performance of the sensor, which limits the measurable spectra (the lowest fluorescence emissions may be found under the noise floor level of the sensor). Thereby, only the highest values of fluorescence emissions, that is, those close to the Chlorophyll a peak placed around the 680 nm, were considered. The second constraint was given by the processing limits of a low-cost hardware. To minimize the signal-processing requirements, algorithms should exhibit an optimal performance in terms of computational cost. In order to fulfill with these two requirements, the signal-processing chain was established in four separated blocks, each one with a different processing function, which include the denoising, the normalization, the transformation and the classification of the input data. The denoising techniques were used to smooth the noisy data, the normalization methods to make the hyperspectral signature of cultures measured at different growth stages equivalent, the transformation block to modify and reduce the data dimension in order to decrease the computational cost while trying to enhance its feature information, and finally the classification algorithms to discern the nature of the samples.

The algorithms of the whole signal-processing chain selected in this research work were experimentally tested and compared using the actual fluorescence measurements obtained from five different species of phytoplankton. In order to deal with the objective of this paper, the data measured with a laboratory spectrofluorometer was synthetically degraded in terms of bandwidth, resolution and signal-to-noise ratio to simulate the performance of a potential low-performance low-cost sensor. Accurate classification results were achieved in most of the combinations when using the original data and, although a decrement in the classification performance was observed; still very good results were obtained in a few combinations when using the degraded quality signal. The optimal chain implementation was obtained by means of the weighted moving average technique as a denoising method, the standard normal variate method for normalization, the principal component analysis to reduce the data dimension and the *k*-neighbors to classify the cultures. The *k*-neighbors does not only provide the best classification results when dealing with fluorescence measurements, but it is also the fastest method, since it does not require a preliminary training, in contrast to the self-organizing maps technique or the growing cell structure method. This combination, additionally, is optimal in terms of the computational cost, and an accurate classification can be achieved with the minimum hardware requirements.

This paper has also confirmed that a suitable discrimination of different taxonomic cultures can be achieved examining only the emission fluorescence data placed around 680 nm and excited at 470 nm, where the fluorescence peak of the Chlorophyll a is allocated. While other research works use the whole visible spectra to classify the samples obtained with accurate but expensive instruments, this work has shown that the 630-to-730-nm band presents enough information to determine the sample origin after smoothing, normalizing and reducing its dimension. This is due to a different proportion of the Chlorophyll-a among different cultures, but also to the presence of different pigment and Chlorophyll-b, -c and -d ratios, which slightly modify the spectral shape of the culture. These results, obtained from fluorescence measurements performed on pure cultures, are a preliminary but necessary validation in order to proceed with the more complex unmixing techniques for taxonomic discrimination in mixed scenarios. The methodology to obtain fluorescence measurements suggests that it is necessary to address this problem using a non-linear unmixing approach (since the photons emitted by one particle can be absorbed or scattered by other particles). This issue opens the way for the development of a wide range of new methodologies and techniques to be implemented in a low-cost instrument, which is of great importance to improve the current lengthy methods of taxonomic identification while reducing the expenses in oceanographic research.

## Figures and Tables

**Table 1.** Algorithms of the four-step signal-processing chain.

**Denoising**	**Normalization**	**Transformation**	**Classification**
WMA	Min-Max	Derivative	*k*-neighbors
Savitzky-Golay	GSM *	Genetic Algorithm *	SOM *
Wavelet *	SNV	PCA	GCS
	Modified SBN *		

* Algorithms modified or developed by the authors.

**Table 2.** Taxonomic groups of the five cultures.

**Species**	**Division**	**Abbreviation**	**Num. of Samples (First Sampling)**	**Num. of Samples (Second Sampling)**
*Thalassiosira weissflogii B*	*Bacillariophyceae*	Thwi	16	22
*Dunaliella primolecta*	*Chlorophyceae*	Duna	18	22
*Pleurochrysis elongata*	*Primnesiophyceae*	Pl	16	22
*Alexandrium minutum*	*Dinophyceae*	Amin	10	16
*Isochrysis Galbana*	*Primnesiophyceae*	Iso	8	22

**Table 3.** Root mean square error (RMSE) between the covariance of the original data and the covariance of the smoothed data at 684 nm.

**Algorithm**	**Parameters**	**RMSE**
WMA (square window)	*n* = 3	0.012
	*n* = 7	0.047
	*n* = 11	0.102
WMA (Gaussian window)	*σ*_1_ = 1.04	0.016
	*σ*_2_ = 1.56	0.030
	*σ*_3_ = 3.12	0.090
Savitzky-Golay	*n* = 13	0.015
	*n* = 17	0.028
	*n* = 23	0.062
Wavelet	*thr*_1_	0.032
	*thr*_2_	0.012

**Table 4.** Kappa indices obtained with the three classification techniques and the four normalization methods when denoising with the weighted moving average (WMA) method (Gaussian window with *σ*_2_).

	**Min-Max**	**GSM**	**SNV**	**SBN**
*k*-neighbors	0.994	**1**	**1**	**1**
SOM	0.925	0.987	**0.994**	**0.994**
GCS	0.974	0.991	**1**	0.974

**Table 5.** Kappa indices obtained with the three classification techniques and the four normalization methods when denoising with the Savitzky-Golay method and *n* = 13

	**Min-Max**	**GSM**	**SNV**	**SBN**
*k*-neighbors	0.994	**1**	**1**	**1**
SOM	0.918	0.987	0.975	**0.994**
GCS	0.965	0.991	**1**	0.982

**Table 6.** Kappa indices obtained with the three classification techniques and the four normalization methods when denoising with the wavelet method (the letter denotes the use either soft or hard threshold).

	**Min-Max**	**GSM**	**SNV**	**SBN**
*k*-neighbors	0.984 s/0.994 h	0.994 s/0.994 h	**1 s/1 h**	**1 s/1 h**
SOM	0.919 s/0.931 h	**1 s**/0.975 h	0.981 s/0.987 h	0.994 s/0.994 h
GCS	0.965 s/0.965 h	**1 s/1 h**	0.991 s/**1 h**	0.982 s/0.965 h

**Table 7.** Kappa indices obtained with the three classification techniques and the three transformation methods, considering the weighted moving average (WMA) (Gaussian window with *σ*_2_) as a denoising method and the modified scale based normalization (SBN) as a normalization method.

	**Derivative (First Order)**	**Genetic Algorithm**	**PCA**
*k*-neighbors	0.994	**1**	**1**
SOM	0.962	**0.994**	0.987
GCS	0.974	0.982	**0.991**

**Table 8.** Computational cost expressed in terms of execution time (in seconds), considering the weighted moving average (WMA) (Gaussian window with *σ*_2_) as a denoising method and the modified scale based normalization (SBN) as a normalization method.

	**Standard**	**Genetic Algorithm**	**PCA**
*k*-neighbors	1.13 s	0.90 s	0.86 s
SOM	19.91 s	14.10 s	13.40 s
GCS	106.06 s	98.10 s	96.87 s

**Table 9.** Averaged confusion matrix obtained with the self-organizing maps (SOM) classification method when using the Savitzky-Golay and the Min-Max methods to denoise and normalize. Results of the confusion matrix have been averaged due to the five-fold cross-validation.

	**Thwi**	**Duna**	**Pl**	**Amin**	**Iso**
Thwi	8	0	0	0	0
Duna	0	8	0	0	0
Pl	0	0.2	7.8	0	0
Amin	0	0	0	8	0
Iso	0.2	0	0	2.2	5.6

**Table 10.** Averaged confusion matrix obtained with the self-organizing maps (SOM) algorithm when using the wavelet (soft threshold) and the Min-Max methods to denoise and normalize. Results of the confusion matrix have been averaged due to the five-fold cross-validation.

	**Thwi**	**Duna**	**Pl**	**Amin**	**Iso**
Thwi	8	0	0	0	0
Duna	0	7.8	0.2	0	0
Pl	0	0	8	0	0
Amin	0	0	0	8	0
Iso	0	0	0	2.4	5.6

**Table 11.** Kappa indices obtained with the three classification techniques and the four normalization methods when denoising with the weighted moving average (WMA) method (Gaussian window with *σ*_2_).

	**Min-Max**	**GSM**	**SNV**	**SBN**
*k*-neighbors	0.750	0.625	**0.994**	0.794
SOM	0.575	0.575	0.943	0.681
GCS	0.279	0.310	0.680	0.517

**Table 12.** Kappa indices obtained with the weighted moving average (WMA) method to denoise, the standard normal variate (SNV) method to normalize, the principal component analysis (PCA) method for a dimensional reduction and the *k*-neighbors to classify, by using the degraded samples with four different variances (*σ*) of the noise Gaussian distribution.

	***σ* = 0.03**	***σ* = 0.06**	***σ* = 0.1**	***σ* = 0.2**
Kappa index	0.981	0.937	0.850	0.787
